
JOURNAL INFORMATION
==============================
NLM Title Abbreviation: Neth Heart J
Journal ID: Neth Heart J
NLM Journal ID: 101095458

Netherlands Heart Journal

ISSN: 1568-5888
EISSN: 1876-6250
Publisher: Springer

ARTICLE INFORMATION
==============================
PMCID: PMC6940407
DOI: 10.1007/s12471-019-01357-1
Article ID: 1357
Article version: 1
Subjects: Thanks to Reviewers

Thanks to reviewers

Electronic publication date: 2019 Dec 13
Print publication date: 2020 Jan
Volume: 28
Issue: 1
First page: 57
Last page: 58
Copyright: © The Author(s) 2019
License: Open Access This article is licensed under a Creative Commons Attribution 4.0 International License, which permits use, sharing, adaptation, distribution and reproduction in any medium or format, as long as you give appropriate credit to the original author(s) and the source, provide a link to the Creative Commons licence, and indicate if changes were made. The images or other third party material in this article are included in the article's Creative Commons licence, unless indicated otherwise in a credit line to the material. If material is not included in the article's Creative Commons licence and your intended use is not permitted by statutory regulation or exceeds the permitted use, you will need to obtain permission directly from the copyright holder. To view a copy of this licence, visit http://creativecommons.org/licenses/by/4.0/.
License URL: https://creativecommons.org/licenses/by/4.0/

issue-copyright-statement: © The Author(s) 2020

==============================
Neth Heart J (2020) · 28:57–58
https://doi.org/10.1007/s12471-019-01357-1

### Thanks to reviewers

Online publiziert: 13 December 2019
© The Author(s) 2019

The Editorial Team would like to extend its gratitude to all reviewers for their time and advice which helped us to assess and improve the papers that were submitted to us in 2019. The reviewers' critical and constructive feedback greatly helped to raise the journal's quality. We would like to Dr. Sebastiaan Bekkers who performed the most reviews in 2019.

Ahmet Adiyaman
Marco Alings
Cor Allaart
Fatih Arslan
Marcel Beijk
Sebastiaan Bekkers
Rypko Beukema
Pranav Bhagirath
Yuri Blaauw
S. M. Boekholdt
Karin Boomars
Bas Boukens
Mohamed Boulaksil
Berto Bouma
Frank Bracke
Carolien Bulte
Kadir Caliskan
Cyril Camaro
Derek Chew
Harry Crijns
Peter Damman
Menko de Boer
Sanneke de Boer
Jonas de Jong
Guus de Waard
Jaap Deckers
Admir Dedic
Peter den Heijer
Dennis den Uijl
Corstiaan den Uil
Dirk Donker
Lukah Dykes
Reinder Evertz
Andreas Gevaert
Sunny Goel
M. Handoko
Magdalena Harakalova
Martin Hemels
José Henriques
R. S. Hermanides
Alexander Hirsch
Niels Hoedemaker
Patrick Houthuizen
Arjan Houweling
Peter Huntjens
Alexander IJsselmuiden
Pieter Jager
Mark Jansen
Harald Jorstad
Luis Eduardo Juarez-Orozco
Otto Kamp
Isabella Kardys
Laura Kerkmeijer
Bas Kietselaer
Jolanda Kluin
Paul Knaapen
Stefan Koudstaal
Roderik Kraaijenhagen
Adriaan Kraaijeveld
T. Krasemann
Narendra Kumar
Tim Leiner
Gerard Linssen
Roberto Lorusso
Joost Lumens
Angela Maas
Alexander Maass
Gabriel Maicas
Olivier Manintveld
Martijn Meuwissen
Firdaus Mohamed Hoesein
Blandine Mondesert
Mohamed Mouden
Hendrik Nathoe
Jan Paul Ottervanger
Sebastiaan Piers
Ron Pisters
Nikki Pluymaekers
Pieter Postema
Saman Rasoul
Michiel Rienstra
Mischa Rijnierse
Danielle Robbers-Visser
Frans Rutten
Bernadet Santema
Nabil Saouti
Simon Schalla
Bastiaan Scholzel
Jaap Seelig
Klaske Siegersma
Cihan Simsek
Krischan Sjauw
Joep Smeets
Sabato Sorrentino
Ruud Spee
Pieter Stella
Bas Streukens
Jurriën ten Berg
Tim ten Cate
Nienke Ter Hoeve
Jonas Teuwen
Ruben Tijssen

### Thanks to Reviewers

Jorik Timmer
Leo Timmers
Pim Tonino
Roderick Treskes
Serge Trines
Floris Udink ten Cate
Wouter van Amsterdam
Ben Van den Branden
Robert van der Boon
Pim van der Harst
Jeroen van der Heijden
René van der Schaaf
Joris van Dijk
Menno van Gameren
Berry van Gelder
Iris van Hagen
Loek van Heerebeek
Kicky van Leeuwen
Jeanine van Lennep
Martijn Stefan van Mourik
Bram Van Wijk
Hugo Verdejo
Niels Verouden
Derk Verschure
Mathijs Versteylen
Maarten Vink
Alexander Vonk
Tom Vromen
Bob Weijs
B. Daan Westenbrink
Sjoerd Westra
Sofieke Wijers
Janneke Wittekoek
Majd Zreik
Bastiaan Zwart
